# Spinal cord stimulation in the approach to chronic pelvic pain: A case report and literature review

**DOI:** 10.1097/MD.0000000000028379

**Published:** 2021-12-30

**Authors:** Estefanía Romero-Serrano, José Miguel Esparza-Miñana

**Affiliations:** Pain Management Unit, Hospital de Manises, Valencia, Spain.

**Keywords:** case report, chronic pelvic pain, experience, review, spinal cord stimulation

## Abstract

**Rationale:**

Chronic pelvic pain (CPP) is a pain related to pelvic structures that arise from posttraumatic pain, postsurgical pain, or somatic or visceral pain syndromes. Despite the available treatment options, CPP remains mostly untreated, due to difficulties in covering such a large area.

**Patient concerns:**

A 46-year-old woman presented chronic low back pain and CPP for more than 1 year and 6 months respectively after multiple pelvic fractures.

**Diagnosis:**

Pelvic fractures and a severe lumbar facet arthrosis were revealed through computed tomography and lumbosacral nuclear magnetic resonance imaging. Evidence of a reduced amplitude in the left femoral nerve and a demyelinating neuropathy in the left pudendal nerve were also detected.

**Intervention:**

A pharmacologic treatment was prescribed, consisting of celecoxib, fluoxetine, gabapentin, and morphine. Since no pain relief was achieved, spinal cord stimulation was performed using spectra WaveWriter system, placing 2 octopolar linear leads over the bilateral T8 and T9 vertebras with the help of a 3-dimensional neural targeting program.

**Outcomes:**

Two weeks after the intervention a reduction of 80% of the pain was achieved, which led to the removal of the pharmacologic treatment. Additionally, both EuroQOL-5D and visual analogue scale scores improved after the intervention.

**Lesson:**

Through the combination of spinal cord stimulation Spectra Wavewriter and 3D programming technology, both lumbar and leg pain and CPP were successfully relieved, along with an improvement in the quality of life of the patient.

## 1 Introduction

Chronic pelvic pain (CPP) refers to continuous pain in pelvis-related structures for at least 6 months. Both men and women are affected^[1]^ with prevalence of 2.7% and 5.7%, respectively. Etiology can include posttraumatic pain, postsurgical pain, or somatic or visceral pain syndromes.^[2]^ Finding the right cause is key to manage the treatment. The pelvic region is innervated by sympathetic and parasympathetic fibers, but also by somatic and splanchnic nerves (lumbar and sacral regions of the spine). The dermatome distribution is T12, L1, L2, and S2, S3.^[2]^

There are several therapeutic options for CPP consisting of physical treatment, psychological therapy, pharmacologic treatment and/or intra-visceral therapies (such as intra-vaginal electrical stimulation or intra-vesical treatments).^[3]^ Interventional pain medicine offers advanced strategies like nerve stimulation, spinal cord stimulation (SCS), or dorsal root ganglion stimulation (DRGS). Despite this variety, CPP is undertreated and mostly keeps any treatment refractory because of the challenge of adequately covering the pain area.

Engineering advances focus on increasing SCS efficacy[^1^,^2^,^3^,^4^] by stimulating the correct target (with anatomically guided 3D neural targeting),^[5]^ and the surge of new devices.

## 2 Case report

A 46-year-old woman suffered multiple pelvic fractures in March 2016. In November 2016 she was referred to the pain unit presenting chronic low back pain for more than 1 year, worse since the accident (vehicle collision), and CPP for more than 6 months. The latter was comprised of a somatic pain localized on the pelvic girdle and a neuropathic pain based on the association of paresthesia in both groins and legs (L2 dermatome on the ventral side). There were also additional lancinating symptoms when urinating. Mean visual analogue scale (VAS) on her baseline pain intensity was 7/10, and increased to 10/10 in movement. The patient presented severe gait problems and a reactive depression. There was a relevant deterioration in the patient's quality of life (QoL).

Computed tomography and lumbosacral nuclear magnetic resonance imaging revealed pelvic fractures (left ilio-pubic branch fracture, a consolidated left ischiopubic branch fracture, an oblique fracture that affected the right ischiopubic branch to the pubic symphysis) in addition to a severe lumbar facet arthrosis. Sphincter abnormalities were not detected. There was evidence of less amplitude in the left femoral nerve, possibly secondary to muscle hypotrophy due to analgesic disuse, and also a demyelinating neuropathy in the left pudendal nerve.

Pharmacologic treatment consisted of the combination of non-steroidal anti-inflammatory (celecoxib 600 mg per day), antidepressant (fluoxetine 30 mg per day), anticonvulsant (gabapentin 1800 mg per day), and oral morphine 90 mg equivalents per day. Pain meant that this therapy remained refractory.

Three caudal epidural blocks were performed, with a reported 20% of pain relief. The patient subsequently underwent infiltration of both third sacral roots and also the ganglion impar with attainment of less than 50% pain relief.

SCS was proposed to the patient in January 2019 to reduce lower lumbar spine pain and neuropathic pain in both legs. Spectra WaveWriter System (Boston Scientific) was selected because of its multiple stimulation waveforms and field shapes.

**Figure 1:**
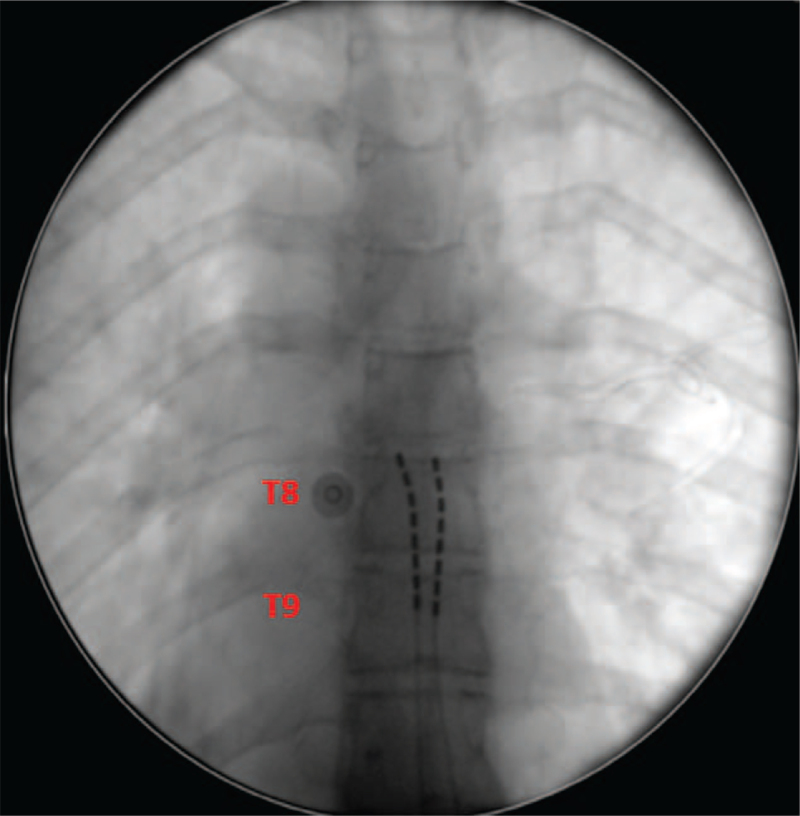
Octopolar linear leads placed over the bilateral T8 and T9.

After psychiatric evaluation, the procedure was performed in April 2019. Access to the posterior epidural space was at L1-L2 level. Two octopolar linear leads were placed over the bilateral T8 and T9 (Fig. 1). Three-dimensional neural targeting programming facilitated the optimal location for leads by attaining appropriate anatomic coverage of the pain area (lower back and legs – dermatome L2 ventral side).

After 2 weeks, the patient achieved 80% pain relief, even with CPP symptoms. Opioids were also withdrawn. The patient was implanted with the leads, which were placed in the same location.

At the time of writing this manuscript, the patient was approximately 18 months postimplant. VAS was reported as 3/10 and several medications have been weaned off.

Her QoL was also assessed using the EuroQOL-5D (EQ-5D) test. In 2016, the EQ-5D resulted in –0.0757, meaning “worse than death”. Nowadays, the EQ-5D value is +0.6454, and the patient reports 80% pain relief (VAS 3/10). Medical treatment has been weaned off, and QoL improved.

**Figure 2:**
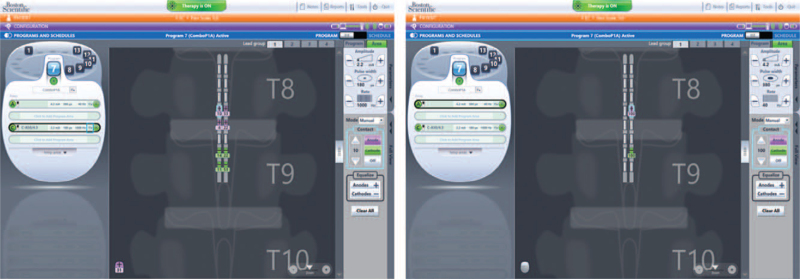
Spectra system programs.

The patient selected program number 7 (Fig. 2) from 16 programs provided by the Spectra system. Therapy consisted of a tonic stimulation whose paresthesia covers the entire area of pain including the lower back, pelvic region, and both legs. This program was combined simultaneously with a 1000 Hz contour high frequency tonic stimulation.

## 3 Discussion

The economic impact of CPP is approximately $8.8 billion in the US. Prevalence ranges from 5.7% to 26.6% in the whole population and is higher in women (9 million in the US).^[4]^ QoL is altered and the incidence of reactive depression and anxiety is more than 38% higher.^[4]^ Pain management is noted for its late diagnosis; 25% of cases are treated by specialists after 3 or 4 years of pain.^[3]^

In regard to this case, pelvic anatomy is very complex. Dermatomes are not as predictable, and pain in the pubic and groin region irradiated to the perineum would involve not only T12, L1, and L2, but also S2 and S3 (Table 1).^[3]^

**Table 1:** Innervation.

Nerve pain	Vertebra location
Iliohypogastric	T12-L1
Ilioinguinal	L1
Genitofemoral	L1-L2
Obturator	L2-L4
Posterior femoral cutaneous	S1-S4
Inferior rectal	S2-S4
Pudendal	S2-S4
Coccygeal	S4-S5

The variety of etiologies and complicated anatomy in the pelvic region[^2^,^3^] makes it difficult to recognize where to find the lead target for therapies, which can cause management to fail. It is important to note that CPP is deemed an entity related to complex regional pain syndrome (CRPS)^[3]^ due to the neuropathic pain reported in both cases, produced by central sensitization. However, CPP and CPRS could also be similar diagnoses because of autonomic dysregulation.

There are several interventional neuromodulatory therapies and pain techniques such as peripheral nerve stimulation, peripheral nerve field stimulation, dorsal nerve root stimulation, sacral neuromodulation, conus medullaris stimulation, transcranial stimulation, and DRGS. They are the most effective treatments for CPP despite possible complications also being reported.[^1^,^3^,^4^]

The dermatomal distribution of the pelvic region (T12-S4) makes it difficult to stimulate a specific region and fibers travel along the autonomic nervous system between T2-L2.

DRG is the intersection of somatic sensory fibers and sympathetic afferences. Stimulating the correct DRG affects both these pathways.

In 2016 the Food and Drug Administration approved this technique for CRPS type 1 and 2 treatment of the lower limb, considered to be similar to CPP. This justifies DRGS possibly being a correct approach for CPP.

CRPS pathophysiology explains how peripheral nerve injury leads to a cascade of events that develop in hyperexcitability and ectopic focus of the upstream cell bodies within the DRG. Central sensitization is promoted and becomes neuropathic pain.

The high selectivity of electrodes to stimulate very specific targets could be a key aspect to account for the benefits attained. Moreover, the electrode has contact with cerebrospinal fluid, which reduces the stimulation's conductivity and effectiveness.

When DRGS and SCS are compared, targets are used to be specifically selected with DRGS. SCS lead location is more variable, but it is possible to capture adjacent fibers by altering the electrical field's parameters.

SCS is becoming increasingly sophisticated by means of the design of implantable pulse generators. In the case reported in this manuscript, Boston Scientific Spectra WaveWriter was the system selected because it enabled the patient to choose from different pre-configured programs with variable stimulation waveforms. Patients can use the programs as needed and simultaneously. Moreover, this SCS was programmed using Illumina 3D programming technology, which is proven through the LUMINA study^[5]^ to be superior to traditional SCS programming. Moreover, it helps to find the optimal target and perfect location for the leads and integrates an algorithm in accordance with the spinal column's electrical conductivity.

Simultaneous use of multiple waveforms resulted in a novel and useful therapy. The painful area was overlapped with paresthesia but was synchronous to high frequency.

## 4 Conclusion

In conclusion, despite CPP use being refractory for non-neuromodulation therapies and SCS having serious difficulties, this manuscript reports successful results using SCS Spectra Wavewriter, a stimulator characterized by different waveforms and types of frequencies which patients are able to select and use simultaneously. However, lead placement still requires further research. The case reported has a different lead location at T8-T9, combined with Illumina 3D Programming Technology to identify optimal targets, where good results were achieved not only for lumbar pain and both legs but for CPP as well. VAS reduced from 10/10 to 3/10, pharmacologic treatment (especially opioid) was withdrawn, and QoL tested by EQ-5D improved.

## Author contributions

**Conceptualization:** Estefania Romero Serrano, José Miguel Esparza-Miñana.

**Methodology:** Estefania Romero Serrano, José Miguel Esparza-Miñana.

**Writing – original draft:** Estefania Romero Serrano, José Miguel Esparza-Miñana.

**Writing – review & editing:** Estefania Romero Serrano, José Miguel Esparza-Miñana.
